# The Use of Pulsed Electromagnetic Fields to Promote Bone Responses to Biomaterials* In Vitro* and* In Vivo*

**DOI:** 10.1155/2018/8935750

**Published:** 2018-09-03

**Authors:** Carlo Galli, Giuseppe Pedrazzi, Monica Mattioli-Belmonte, Stefano Guizzardi

**Affiliations:** ^1^Dep. of Medicine and Surgery, University of Parma, Italy; ^2^DISCLIMO, Department of Clinical and Molecular Sciences, Polytechnic University of Marche, Ancona, Italy

## Abstract

Implantable biomaterials are extensively used to promote bone regeneration or support endosseous prosthesis in orthopedics and dentistry. Their use, however, would benefit from additional strategies to improve bone responses. Pulsed Electromagnetic Fields (PEMFs) have long been known to act on osteoblasts and bone, affecting their metabolism, in spite of our poor understanding of the underlying mechanisms. Hence, we have the hypothesis that PEMFs may also ameliorate cell responses to biomaterials, improving their growth, differentiation, and the expression of a mature phenotype and therefore increasing the tissue integration of the implanted devices and their clinical success. A broad range of settings used for PEMFs stimulation still represents a hurdle to better define treatment protocols and extensive research is needed to overcome this issue. The present review includes studies that investigated the effects of PEMFs on the response of bone cells to different classes of biomaterials and the reports that focused on in vivo investigations of biomaterials implanted in bone.

## 1. Biomaterials and Bone Regeneration

Biomaterials play an important role in bone regenerative strategies [1] in both orthopedics and dentistry as scaffolds [2] or as a support for prosthesis, e.g., hip or dental implants [3]. In all these clinical situations the challenge biomaterials must face is to integrate in the host and promote bone healing along its surfaces [4], albeit with noticeable differences. Most scaffolds are made of resorbable materials, because common opinion dictates that scaffolds should progressively be replaced by native tissue [5], whereas prostheses are mostly permanent implants and their purpose is to last and function as long as possible in patients, usually while withstanding relevant mechanical forces in the process [6]. Thus, most scaffolds currently used in bone are made of bioceramics, predominantly calcium phosphates, because of their chemical similarity to the inorganic matrix of bone [7], which makes them osteoconductive [8, 9]. Furthermore, bioceramics are rigid and their mechanical properties have been shown to positively affect cell differentiation along the osteoblastic lineage [10, 11]. Last but not least, this class of biomaterials is usually very biocompatible and resorbable within a time span that appears to quite closely meet the requirements for implantation into natural bone [8]. Although bioceramics can be loaded with biologically active ions [12] or biomolecules [13] to improve bone formation, they are not as versatile and customizable as polymers, whose structure can be modified almost* ad libitum*, enabling researchers to add functional groups and control their polymerization, their chemical behavior, their mechanical properties, and resorbability [14–16]. Polymers have opened up hitherto unexplored possibilities, such as injection of photopolymerizable compounds [17] or easy 3D printing [18].

In contrast, implantable prostheses are still mostly made of titanium and its alloys, although novel and highly resistant ceramics, i.e., zirconia, could represent a viable alternative [19, 20]. Titanium is a very biocompatible metal, which has been shown to represent an efficient material for orthopedic and dental implants [21]. A lot of effort has gone into investigating optimal surface treatments to optimize bone response and speed up tissue healing after surgery [4, 22]. What bioceramics, most polymers, and metals still lack is, however, specific biochemical cues that can control cell behavior toward desired clinical goals, beside generic stimuli, such as calcium release from resorbable bioceramics or stiffness-related mechanical stimulation of cell differentiation, unless of course these materials are loaded with bioactive compounds [13, 23]. Most of these materials still offer the organism just a viable framework within which to heal or regenerate, supporting the process but fundamentally relying on the drive to healing that is intrinsic to many tissues, especially epithelia and bone. This means that those numerous clinical situations where the tissue regenerative potential has been compromised due to age or pathology are still a serious challenge and adjunctive or ancillary therapies are still an issue of interest and hot debate. This is where additional, physical therapies such as electromagnetic fields could play an important, if not vital, role.

## 2. Electromagnetic Fields and Bone

Electromagnetic fields (EMF) are created by the interaction of electrically charged objects and permeate our whole reality [24]. Our world is flooded with artificial EMFs created by electrical and electronic devices [25] and although these have become a source of potential health concerns [26–31], research has long sought a way to harness their therapeutic potential [32]. To this purpose, different sources of low frequency EMFs have been actively investigated. These can be further divided into Pulsed EMFs (PEMFs), where the EMF signal is delivered in pulses of different shape interspersed with gaps and sinusoidal EMFs (SEMFs), where the superposition of the EMF signal continuously and gradually varies along a sine waveform [33].

It is known that the effects of electromagnetic fields on living beings are complex. Organisms are composed of cells, which possess an electrically charged membrane and tightly regulate the concentration of ions, electrically charged particles, e.g., Ca^2+^ or Na^+^, which they use as potent signal mediators [34]. It is therefore likely that most of the effects of EMFs in cells occur or are triggered at the membrane level. There is abundant evidence suggesting that EMFs can act on Ca^2+^ concentration [35–37] and Ca-dependent pathways [38], and more recently Vincenzi et al. have convincingly shown a regulation of Adenosine receptors by PEMFs [39]. Actually the recent evidence by Yan et al. [40] and Xie et al. [41] of a role of primary cilia in transducing EMF effects in cells could be a part of a broader activity on membrane trafficking, including receptor trafficking. Further mechanisms are likely to be involved as PEMFs have been shown to modulate defenses against Reactive Oxygen Species [42] and the production of bioactive factors [40, 43–45] and to activate intracellular pathways such as the sAC–cAMP–PKA–CREB signaling pathway [46].

Most life science and biomedical research has been focused on the biological effects of PEMFs of different waveform, frequency, and intensity on different tissues and in different clinical situations. Bone has long been recognized as a suitable target for EMF treatment [47].

Indeed EMFs have been investigated as a tool to promote bone healing in several preclinical studies of bone defect healing in rodents, encompassing diverse defect models, e.g., limb or facial defects [48–56], bone loss due to (a) hyperparathyroidism [57], (b) glucocorticoids or ovariectomy [58–66], (c) disuse [67–69], or (d) diabetes [70], or even osteoporotic fractures [71] or osteoarthritis [72]. Different animal models, e.g., horses, were used as well for PEMF testing [73, 74], with positive results.

EMFs have also a long clinical story as an aid to reduce bone loss in osteoporosis [75–77], to improve osteotomies or nonunions [78–93], and different research groups have investigated frequencies, intensities, durations of exposure, pulses [94–97], or waveforms [98].

Actually EMFs can be administered in a vast range of modalities. Stimuli can be delivered as single pulses, or discrete pulses, or even complex arrays of pulse bursts, also known as Pulsed Radio Frequencies (PRF), similarly to FM radio receivers. In this case the single pulses that constitute the carrier frequency reach the kHz range, but these are modulated into sets or trains of pulses that cycle at slower frequency, often 15 Hz. Using high carrier frequency increases the penetration of EMFs throughout the body, which then is able to demodulate the signal and perceive the modulating frequency, which exerts the biological effect [99]. Intensities range across a wide spectrum as well, from *μ*T to a few mTesla. However, a fundamental lack of understanding of the mechanisms of actions of EMFs on cells and tissues has been presented to reach a consensus on a set of clinical parameters to maximize the effects of EMFs [47].

To further compound this problem, it must be remembered that different biomaterials may require different stimulations to optimize the outcome and this has also hindered proving their clinical effectiveness, in spite of promising results [100–103].

Therefore, the present study will review the available literature on the effects of EMF treatment on osteoblasts and bone* in vitro* and in preclinical animal models* in vivo*.

## 3. The Effects of PEMFs on Osteoblasts

Several parameters have been shown to affect cell responses, e.g., PEMF waveform, its frequency, its intensity, or the duration of exposure. A study by J. Zhou et al. investigated the effects of EMF waveform on primary rat calvaria cells [98]. When comparing 50 Hz, 1.8 mT sinusoidal, triangular, square, or serrated EMFs on primary osteoblasts, the authors observed that only square waves significantly increased cell proliferation and that sinusoidal waves decreased it. Interestingly, only triangular and sinusoidal waves, however, significantly increased cell differentiation, as assessed by Alkaline Phosphatase activity or mineralization assays. Although the group by Zhang et al. reported similar findings [33], other studies report conflicting evidence.

Martino et al. [104] exposed human osteosarcoma SaOS-2 cells to 0.9 mT, 15 Hz PRF PEMF quasi square bursts of 4 kHz square pulses for 4 hours/day, and they observed an increase in ALP activity and the deposition of mineralized nodules although no effect on cell proliferation was reported. Their results were confirmed by Hannay et al., who applied a similar stimulation (15 Hz PRF bursts of trapezoidal pulses) with a 1.6 mT intensity to Saos-2 and observed significant increase in ALP activity [105]. Other cell models, such as human osteosarcoma MG-63 [43, 106–108], mouse calvaria osteoblastic cell line MC3T3-E1 [36, 95, 109–114], rat primary calvaria cells [37, 40, 41, 45, 115, 116], primary human osteoblasts [42, 117–119], adipocyte-derived mesenchymal stem cells [118, 120–122], or bone marrow stromal cells [120, 123–133] were tested as well. As anticipated, most studies on osteoblast-related cell models rely on the 50-75 Hz range of stimulation [40, 41, 107, 108, 134–137] or, alternatively, on the use of 15 Hz PRF burst system [43–45, 105, 111, 112, 132, 138, 139]. The spectrum of intensities used is quite broad but, taken together, most works focus on the 0.6-2 mT [40, 41, 110, 137].

When osteoblastic cells grow on biomaterials however, a further layer of complexity is added. For the sake of simplicity, these studied were divided according to the nature of the biomaterial used.

## 4. PEMFs and Calcium Phosphate Scaffolds

All the studies on EMFs and calcium phosphate scaffolds included in the present review are listed in Table 1. One of the first studies to investigate the effects of PEMFs on bone response to bioceramics was performed by Shimizu et al. who implanted porous hydroxyapatite (HA) or tricalcium phosphate (TCP) cylinders in the proximal tibia of rabbits, which were then exposed to 1.5 Hz, 26 ms-long PFR PEMF bursts at 0.18 mT intensity for 8 hours/day. They were able to demonstrate a beneficial effect of PEMF stimulation on bone ingrowth into HA samples, with a higher amount of newly formed bone in and around HA, in both the cortical and medullary area, up to 4 weeks after surgery, but not around TCP implants [140]. A morphological evaluation of bone ingrowth into natural or synthetic hydroxyapatite granules implanted into rabbit tibia defects was conducted by Ottani et al. using 50 Hz triangular-shaped PEMF pulses at an intensity of 8 mT for 30′-long sessions twice a day. The sacrifice and subsequent TEM and SEM observation with electron backscattering at 2 and 4 weeks after surgery showed that PEMF treatment promoted a more advanced bone formation around the granules, which appeared cemented into the healing defect [141]. In the same year a study by Fini et al. was published, which investigated the effects of PEMFs on the integration of synthetic HA rods obtained by granule sintering in bone defects created in rabbit femoral condyles. The group used 1.35 ms-long trapezoidal PEMF pulses, repeated at a 75 Hz frequency, with an intensity of 1.6 mT for 6 hours/day for 3 weeks. Although histomorphometry did not reveal any increase in bone architectural parameters after PEMF stimulation at either 3 or 6 weeks after surgery, the bone-to-implant contact (BIC) was increased in the PEMF-treated group at both time points. The same happened with the mechanical properties of the treated bones, as assessed by hardness to microindentation [142]. The same research group adopted this stimulation model again to evaluate the integration of synthetic HA rods in the cortical bone of rabbit femurs and observed that PEMFs were able to significantly increase bone-to-implant contact, Mineral Apposition Rate (MAR), and Bone Formation Rate (BFR) at both time points. They also confirmed that the mechanical properties of treated bones were increased by PEMFs, using both indentation and push-out tests [143]. The cellular effects of PEMFs on the response of human Saos-2 osteosarcoma cells to discs of porous bovine natural apatite were investigated by Fassina et al., who exposed cells to 1.3 ms trapezoidal pulses at 75 Hz, 2 mT in bioreactors for 24 hours/day for 22 days [144]. In response to PEMFs the authors observed an increase in cell proliferation and the deposition of components of the extracellular matrix.

The group by Schwartz et al. investigated the effects of electromagnetic fields on human mesenchymal stem cells, using an established stimulation model of 4.5 ms PEMF bursts at 15 Hz frequency, with each burst composed of 225 *μ*s-long pulses. Cells were grown on commercially available calcium phosphate discs and were exposed to PEMFs for 8 hours/day. Although, in their model, they did not observe significant effects of PEMFs on cell number or differentiation markers, the group found that electromagnetic fields synergistically stimulated cell responses to BMP-2 and promoted Alkaline Phosphatase (ALP) activity, Osteocalcin expression, and the release of TGF*β*1 [145].

Interestingly, BMPs have been shown to be involved in the responses of rat calvaria osteoblasts to PEMFs in a study by Bodamyali et al. [45] and by Yan et al. [40]. Selvamurugan et al. demonstrated that PEMFs and BMP-2 may act synergistically in rat osteoblasts and this could be indicative of similar or overlapping signaling pathways in bone cells [115]. The group by Schwartz et al. also investigated the response of mesenchymal stem cells, commercially available normal human osteoblasts, or osteoblastic cells from two well established cell lines (MG-63 and Saos-2 cells) to 8-hour long exposures to 4.5 ms-long pulse bursts repeated at 15 Hz [146]. Their study showed that PEMFs were able to increase OPG expression in cell lines when cultured on calcium phosphate discs and synergistically increase OPG when administered together with BMP-2 in mesenchymal stem cells, while not affecting RANKL. Given the relevance of the OPG-RANKL system in bone, the effects of PEMFs on these molecular effects have been extensively studied in several osteoblastic models, also in the absence of biomaterials, and most studies agree with the results from Schwartz's groups in observing an increase in OPG following PEMF exposure. This is of obvious interest to bone researcher, because of the role of OPG and RANKL for tissue metabolism [147–150]. Schwartz's results were confirmed in cell cultures on plastic by Borsje et al. and similarly by Jansen et al. using BMMSCs [129] and even in human marrow macrophages cultures [132]. The group by Chang et al. showed that 7.5 Hz 0.3 ms long PEMF pulses increased OPG secretion [151] in mouse bone marrow cells [151]. They also observed that PEMFs enhanced OPG and hampered RANKL expression in mouse primary calvaria cells [152].

## 5. PEMFs and Titanium Surfaces or Implantable Devices

The effects of PEMFs on metal devices have been investigated in several studies. Though stainless steel implants in rabbit tibia and femurs were investigated by Spadaro et al., who observed an increase in the amount of formed bone in the medullary canal of femurs around moveable steel wires after 15 Hz PRF PEMF stimulation [153], most of the subsequent research focused on titanium and titanium alloy-based biomaterials. Saos-2 cells were used as a model of osteoblastic cells on titanium fiber-mesh scaffolds and continuously stimulated with 1.3 ms trapezoidal pulses at 75 Hz, 2 mT in bioreactors for 22 days. It was shown that PEMFs increased the expression of TGF-*β* and upregulated the deposition of matrix on the scaffolds, by increasing the expression of Decorin, Osteopontin, and Type I collagen [154]. The same group investigated the effects of PEMFs using the same cell and stimulation model on sintered titanium grids [155], observing similar findings. Wang et al. stimulated primary rat calvaria cells with 15 Hz, 5 ms long bursts of 4.5 kHz pulses, 0.9 mT, on polished, sand-blasted/acid-etched or anodized nanotubular titanium surfaces [156]. Interestingly, PEMF stimulation increased protein adsorption and cell adhesion on all titanium surfaces, cell proliferation up to 7 days, and cell mineralization on all surfaces. PEMF also affected cell morphology and induced more pseudopodia and cytoskeletal reorganization that aligned cells along their main axis. Interestingly, PEMFs also increased BMP-2 expression, beside differentiation markers. Bloise et al. [157] recently stimulated human BMMSCs nanostructured TiO_2_ surfaces obtained through cluster-assembly by a pulsed microplasma cluster source [158, 159] with 1.3 ms long, 75 Hz PEMFs at 2 mT intensity for 10 min/day. The authors observed an increase in osteogenic differentiation in PEMF-stimulated cells, an increase in the intracellular levels of Ca^2+^, and an increase in the extracellular Ca^2+^ deposition.

Using TiZr or titanium discs with different topography, Atalay et al. showed that the proliferative response of primary calvaria cells to 100 Hz PEMFs was clearly dependent on the microgeometry and physicochemical properties of the substrate [160].

The group of Jing et al. used 15 Hz, 5 ms long PEMF bursts with 2 mT intensity to stimulate MC3T3-E1 cells on porous titanium scaffolds (70% porosity, 750 *μ*m pore size) for 2 hours/day for 3 days [161]. Besides observing an increase in cell proliferation and expression of differentiation markers Runx2 and Osterix, two important transcription factors activated in osteoblasts, the group reported that PEMF treatment increased *β*-catenin, Lrp6, and Wnt1 expression, important components of the canonical Wnt pathway, at the mRNA and protein levels. Remarkably, these findings were confirmed in vivo after implanting porous titanium scaffolds in cylindrical defects in the femur of rabbits, which were then treated for up to 12 weeks with PEMFs. MicroCT analysis of the defects showed that PEMF treatment significantly improved bone architectural parameters, e.g., BV/TV, Trabecular Number (Tb.N), and spacing (Tb.Sp), and dynamic histomorphometry demonstrated that MAR, Mineralizing Surface, and BFR were significantly higher in rabbits treated with PEMFs than control animals. Moreover, real time PCR indicated an increase in the expression of BMP-2, consistently with Lohmann [145, 146], but also Wnt1, Lrp6 and *β*-catenin as observed* in vitro*.

These results are in agreement with Single Pulsed EMF (sPEMF) exposure of MC3T3 cells on plastic culture substrates [114]. The authors exposed this cell line to 0.2 Hz, 5 ms long, 1 T PEMF pulses for up to 20 days and observed an increase in the expression of Wnt1, Wnt3a, Wnt10b, and Wnt receptor frizzled 9 and an increase, albeit not significant, of the Wnt coreceptor Lrp6. Similarly, Zhai et al. [110] observed that 2 mT, 15 Hz bursts of 4.5 kHz PEMF pulses for 2h/day for 3 days increased the expression of Wnt1, Lrp6, and *β*-catenin in MC3T3-E1 cells.

Buzzà et al. used 85 *μ*s long pulses at 20 MHz for 30′/day for up to 42 days to stimulate titanium implants in rabbit tibias but failed to observe any significant increase in removal torque [162]. A slightly lower PEMF frequency (1 MHz, 25 *μ*s long pulses, 0.8 mT) was used by do Nascimento et al. for 20′/day for 2 weeks to stimulate postextractive dental implants in dog mandibles. The authors observed a slight increase in bone tissue formed around the implants, although no quantification was provided [163]. Matsumoto et al. investigated the effects of 100 Hz, 25 *μ*s PEMFs at 0.2, 0.3, or 0.8 mT for 4 or 8 hours/day on the integration of Ti-6Al-4V dental implants with anodized surface into rabbit femurs and reported that BIC was higher after exposure to 0.2 or 0.3 mT PEMFs for 4 or 8 hours [164]. This stimulation model was also used with Ti-6Al-4V dental implants inserted in rabbit mandibles. The animals were stimulated with PEMFs for 2 weeks and sacrificed right after 2 weeks or 6 more weeks (without PEMF application). Remarkably, although no differences were observed at 2 weeks and 6 weeks after PEMF stimulation a dramatic increase in labial and lingual bone was observed in treated animals, together with higher osteoblast counts, indicating that PEMF could promote a long-acting bone formation [165]. A similar PEMF stimulation model was used by Akca et al. to investigate the effects of PEMFs on the integration of cylindrical titanium implants in tibias of ovariectomized rats. The animals were stimulated for 4 hours/day for 14 days and PEMF stimulation increased Bone Volume and trabecular number in the peri-implant bone, as determined by microCT [166]. A study by Grana et al. investigated the effects of 60 ms, 1.9 Hz PEMF bursts of 50 Hz sinusoidal trains at an intensity of 72 mT administered for 30′/twice a day on bone healing around titanium mini implants in rat tibias and found a significant increase in the amount of newly formed bone around implants at 10 and 20 days after surgeries [167]. Ten Hz, 0.4 mT PEMFs were investigated as a tool to improve the bone integration of commercially available titanium dental implants inserted in rabbit tibias in a more recent study [168]. Most noticeably, PEMFs were generated by a portable device which was installed on the implant, via a screw-retained connection, not unlike common prosthetic components. The device generated a magnetic field that was concentrated around the coronal area of the implant and steeply decreased in the surrounding areas. When considering the coronal area alone, where the signal was stronger, Bone Volume/Total Volume around test implants was 56% and 68% significantly higher than control implants at 2 and 4 weeks of healing, respectively, with corresponding increased Tb.N and smaller Tb.Sp. Moreover, by 2 weeks BIC was 15% higher around stimulated implants [168]. The idea of installing intraoral devices to stimulate implants with PEMFs was explored in several papers, as devices generating 10 Hz PRF PEMF bursts at 2 mT were proposed [169] (or even neodymium-iron-bore magnets placed in the implants and generating static magnetic fields [170]). Twenty-five *μ*s PEMFs at 10 Hz and 0.2 mT were also investigated as a tool to promote the integration of porous titanium implants in the diaphysis of rabbit humerus bones for 5 or 10 hours/day and shown to increase bone ingrowth by a 14-day stimulation [171]. Cai et al. showed that 15 Hz, 5 ms PEMF bursts of 4.5 kHz pulses 2 hours/day for 8 weeks improved bone turnover serum markers and bone architecture parameters in rabbits with alloxan-induced type 1 diabetes mellitus (T1DM). More importantly for our current review, when cylindrical sintered Ti2448 implants were inserted into the lateral condyle of these rabbits, the 8-week treatment improved bone ingrowth into the scaffold and MAR around and inside the implants, which caused an increase in the mechanical properties of the trabecular bone around the implants [172]. For a list of the studies on EMFs and titanium biomaterials included in the present review, please see Table 2.

## 6. PEMFs and Polymers

Table 3 summarizes all the studies on polymer scaffolds and EMFs that were included in the present review. Polymer scaffolds were tested for cell responses to PEMFs as well. Electrospun poly(caprolactone) nanofibrous scaffolds were used as substrate to culture adipose tissue-derived stem cells, which were then stimulated with 50 Hz, 1 mT PEMFs for 6 hours/day in normal or osteogenic medium [173]. PEMFs increased cell proliferation, mineralization, and the expression of differentiation markers, such as Runx2, Osteocalcin, Osteonectin, and ALP activity. The group of Tsai et al. cultured rat calvaria osteoblasts on highly porous poly(DL-lactic-co-glycolic acid) (PLGA) scaffolds in bioreactors and stimulated them for 2 or 8 hours/day with 300 *μ*s long rectangular pulses at 7.5 Hz. The magnetic field they used had an intensity of 0.13, 0.24, or 0.32 mT. Interestingly, stimulation with 0.13 mT PEMFs was able to significantly increase cell number on the scaffolds up to day 12 of culture, while more intense 0.32 mT PEMFs significantly decreased cell number compared to the control group up to day 18 of culture. However, not surprisingly, the highest intensity was also most effective in increasing ALP activity and thus cell differentiation [174]. Lin et al. used an* in vitro* inflammation model to study the effects of 75 Hz, 1.5 mT PEMFs, using previously well described instrumentation [108] in 7F2 murine osteoblasts cultured on 3D chitosan scaffolds exposed to 9 hours of treatment [135]. The osteoblastic cells were cocultured with LPS-activated RAW 264.7 macrophages. The investigators detected higher Nitric Oxide levels after PEMF treatment, consistently with the previous literature [112, 175, 176], but increased osteoblast viability and collagen expression, although reduced differentiation, as measured by ALP activity and Osteocalcin levels. In agreement with their observations, Ehnert et al. [42] exposed primary human osteoblasts to 16 Hz 0.28 mT PEMF bursts for 7 minutes/day and demonstrated an increase in defenses against reactive oxygen species after PEMF stimulation [119], which actually appears necessary for PEMF effect [42].

The response of human osteosarcoma MG-63 cells to trapezoidal 1.3 ms long, 75 Hz, 2.3. mT PEMF pulses [134] when cultured on poly-methylmethacrylate (PMMA) scaffolds or PMMA-alpha Tricalcium Phosphate (*α*-TCP) composite scaffolds was investigated by Torricelli et al. [177]. Cells were stimulated for 12 hours/day for 3 days, and PEMFs were able to increase the expression of Osteocalcin, C-terminal procollagen type 1, and TGF*β*1 in cells on composite scaffolds, while decreasing IL-6 expression by 6 days of culture. An involvement of TGF-*β* in PEMF stimulation was highlighted by several researches in MG63 cells [43], in serum-starved MC3T3 cells [111], and in human BMMSCs, where PEMFs increased Smad-2 and miRNA21, a microRNA targeting Smad-7, a TGF-*β* signaling inhibitor [131].

Veronesi et al. showed that 75 Hz, 1.5 mT PEMF stimulation for 4 hours/day improved 40-day healing in osteochondral defects in rabbit knees, when used together with collagen scaffolds [178]. Collagen sponges loaded with increasing doses of recombinant human BMP-2 were also implanted in calvaria defects in rats and treated with 1 mT, 60 Hz PEMF stimulation for 8 hours/day for 5 days [179]. Computer microtomography 4 weeks after surgery revealed that PEMF stimulation increased Bone Volume and Bone Mineral Density in the absence or in the presence of rhBMP-2 but not with the highest, 10 *μ*g, dose, where no additional effect was observed. In the samples implanted with 2.5 micrograms as well PEMF stimulation significantly increased also Tb.N. and decreased Tb.Sp. Similarly, histology showed that PEMFs were able to increase bone regeneration in the central area of the defect without the addition of rhBMP-2.

Hydrogels were also explored together with PEMF exposure. Fassina et al. [180] cultured Saos-2 cells in bioreactors on methacrylamide-modified gelatin type B using the same exposure model as previously described [144, 181] and observed an increase in the deposition of Extracellular Matrix. Some research groups are also creating EMF-responsive hydrogels, which can release their bioactive load under EMD stimulation, e.g., methacrylated chondroitin sulfate (MA-CS) hydrogels coated with iron-based magnetic nanoparticles for PDGF release [182] and Ca^2+^-crosslinked Alginate/Xanthan gum hydrogels with magnetite particles for dopamine delivery [183, 184], although these studies were not included in the present review as EMFs were used only as a release-triggering stimulus and not to elicit biological effects.

## 7. Conclusions

The world of biomaterials is as diverse as the clinical applications that rely on them; therefore it stands to reason that there is no easy solution to improve their performance and the responses of the organisms to implanted material and devices. We nevertheless attempted at simplifying the wealth of available materials by dividing them into three main categories, which are however broad as well. A few conclusions can be drawn.

PEMFs have been repeatedly shown to possess the potential to affect osteoblast behavior on different biomaterials and thus represent a potential tool to improve the clinical outcome of several regenerative and prosthetic therapies in orthopedics and dentistry and should be more thoroughly investigated by proper clinical trials.

The response of cells and tissues to PEMF in the presence of titanium devices, for orthopedic or dental use, has been investigated using a vast range of PEMF approaches and settings but besides a few attempts in the early 2000s with 100 Hz PEMF pulses with very light intensities, around 0.2 mT (following the seminal work by Matsumoto et al. [164]), most recent studies are narrowing down their focus to 15 Hz PRF PEMF stimulation or 75 Hz trapezoidal stimuli, with higher intensity, around 1-2 mT. Similar conclusions can be achieved considering the biological responses to bioceramic and polymer scaffolds. However broader screening studies testing cell or tissue responses across a spectrum of frequencies are still missing, though they would be sorely needed to better understand and possibly overcome the differences that exist among schools, with the purpose of establishing better and more reliable clinical protocols for this powerful technology.

## Figures and Tables

**Table 1:** The table summarizes the in vitro and in vivo studies on the effects of PEMF stimulation on osteoblastic primary cells and cell lines on calcium phosphate biomaterials. Studies are listed in chronological order.

**Experimental model**	**Biomaterial**	**PEMF**	**Field intensity (mT unless otherwise specified)**	**PEMF waveform**	**Exposure**	**PEMF Generator**	**Reference**
Defects in proximal tibia of rabbits	Porous hydroxyapatite (HA) or tricalcium phosphate (TCP) nails	1.5 Hz, 26 ms-long PEMF bursts of 3.8 kHz pulses	0.18	Quasi square	8 hours/day for up to 6 weeks	American Medical Electronics (Dallas, TX, U.S.A.)	(Shimizu et al., 1988)

Defects in rabbit tibia	Natural or synthetic hydroxyapatite granules	50 Hz	8	Triangular	30 min/12 hours for up to 4 weeks	In-house built generator	(Ottani et al., 2002)

Defects in rabbit femur (condyles)	Synthetic HA rods obtained by granule sintering	1.3 ms-long, 75 Hz	1.6	Trapezoidal	6 hours/day for 3 weeks	BIOSTIM, Igea, Carpi, Italy	(Milena Fini et al., 2002)

Defects in rabbit femurs (cortical bone, mid-diaphysis)	Synthetic HA rods obtained by granule sintering	1.3 ms-long, 75 Hz	1.6	Trapezoidal	6 hours/day for 3 weeks	BIOSTIM, Igea, Carpi, Italy	(M. Fini, Giavaresi, Giardino, Cavani, & Cadossi, 2006)

Commercially available human mesenchymal stem cells	Commercially available calcium phosphate discs	4.5 ms-long, 15 Hz bursts of 4.4 kHz, 225 *μ*s-long pulses	1.6	Quasi-square (with trapezoidal pulses)	8 hours/day	Electro-Biology Inc., Parsippany, NJ	(Z. Schwartz et al., 2008)

Commercially available mesenchymal stem cells, normal human osteoblasts, MG-63 or Saos-2	Commercially available calcium phosphate discs	4.5 ms-long, 15 Hz bursts of 4.4 kHz, 225 *μ*s-long pulses	1.6	Quasi-square (with trapezoidal pulses)	8 hours/day	Electro-Biology Inc., Parsippany, NJ	(Zvi Schwartz, Fisher, Lohmann, Simon, & Boyan, 2009)

Human osteosarcoma Saos-2 cells	Commercially available discs of porous bovine natural apatite	1.3 ms pulses at 75 Hz	2	Trapezoidal	24 hours/day for 22 days	BIOSTIM, Igea, Carpi, Italy	(Lorenzo Fassina et al., 2010)

**Table 2:** The table summarizes the in vitro and in vivo studies on the effects of PEMF stimulation on osteoblastic primary cells and cell lines on titanium-based biomaterials. Studies are listed in chronological order.

**Experimental model**	**Biomaterial**	**PEMF**	**Field intensity (mT)**	**PEMF waveform**	**Exposure**	**PEMF Generator**	**Reference**
Placement in the medullary canal of femur and tibia in rabbits	Implants of 316 L stainless steel wire	5 ms, 15 Hz PEMF bursts of 4 kHz pulses	n/a	Quasi-square (trapezoidal pulses)	4 hours/day for 2 weeks	American Medical Electronics (Dallas, TX, U.S.A.)	(Spadaro et al., 1990)

Diaphysis of rabbit humerus	Bead-covered titanium implants	25 *μ*s PEMF pulses at 10 Hz	0.2	n/a	5-10 hours/day for 2 weeks	n/a	(Ijiri et al., 1996)

Placement in rabbit femurs	Commercially available Ti-6Al-4V dental implants with anodized surface	100 Hz, 25 *μ*s PEMFs	0.2, 0.3, 0.8	n/a	4 or 8 hours/day for up to 4 weeks	Riken Electromagnetic Field Pulse Generator, Institute of Physical and Chemical Research, Saitama, Japan	(Matsumoto et al., 2000)

Placement in rabbit tibias	Commercially available titanium dental implants	85 *μ*s-long pulses at 20 MHz	1 W	n/a	30 minutes/day for 21 or 42 days	Healtec-Celular, Healtec Eletromedicina Ltd., Brazil	(Buzzá et al., 2003)

Placement in rabbit mandibles	Custom Ti-6Al-4V dental implants	100 Hz, 25 *μ*s PEMFs	0.2	n/a	4 hours/day for 14 days	In-house built	(Özen et al., 2004)

Placement in tibias of ovariectomized rats	Cylindrical titanium implants	100 Hz, 25 *μ*s PEMFs	0.2	n/a	4 hours/day for 14 days	In-house built	(Akca et al., 2007)

Human osteosarcoma Saos-2 cells	Titanium fiber-mesh sheets	1.3 ms pulses at 75 Hz	2	Trapezoidal	24 hours/day for 22 days	BIOSTIM, Igea, Carpi, Italy	(Fassina et al., 2008b)

Human osteosarcoma Saos-2 cells	Sintered titanium grids	1.3 ms pulses at 75 Hz	2	Trapezoidal	24 hours/day for 22 days	BIOSTIM, Igea, Carpi, Italy	(Fassina et al., 2008a)

Placement in rat tibias	Custom cylindrical threaded titanium implants	60 ms, 1.9 Hz PEMF bursts of 50 Hz trains	72	Quasi-square (with sinusoidal pulses)	30 minutes/twice a day	Magnetherp (Meditea Electromédica, Buenos Aires, Argentina)	(Grana et al., 2008)

Dog mandibles, immediate post-extraction placement	Commercially available titanium dental implants	1 MHz, 25 *μ*s-long pulses	0.8	n/a	20 minutes/day for 2 weeks	n/a	(do Nascimento et al., 2012)

Primary rat calvaria cells	Commercially pure titanium or TiZr discs	100 Hz, 25 *μ*s PEMFs	0.2	n/a	2 hours/day for up to 72 hours	In-house built	(Atalay et al., 2013)

Primary rat calvaria cells	Polished, sand-blasted/acid-etched or anodized nanotubular titanium surfaces	15 Hz, 5 ms-long bursts of 4.5 kHz pulses	0.96	Quasi-square (with square pulses)	Up to 7 days	GHY-III, FMMU, Xi'an, China	(Wang et al., 2014)

Placement in rabbit tibias	Commercially available titanium dental implants	10 Hz	0.4-0.2	n/a	24 hours/day for 2 or 4 weeks	n/a	(Barak et al., 2016)

Murine MC3T3-E1 osteoblastic cells	Porous titanium scaffolds by electron beam melting system	15 Hz, 5 ms-long bursts of 4.5 kHz pulses	2	Quasi-square (with square pulses)	2 hours/day for 3 days	GHY-III, FMMU, Xi'an, China	(Jing et al., 2016)

Defects in rabbit femurs (condyles)	Porous titanium scaffolds by electron beam melting system	15 Hz, 5 ms-long bursts of 4.5 kHz pulses	2	Quasi-square (with square pulses)	2 hours/day for 6 or 12 weeks	GHY-III, FMMU, Xi'an, China	(Jing et al., 2016)

Placement in rabbit femurs (condyles)	Cylindrical sintered Ti2448 implants	5 Hz, 5 ms PEMF bursts of 4.5 kHz pulses	2	Quasi-square (with square pulses)	2 hours/day for 8 weeks	GHY-III, FMMU, Xi'an, China	(Cai et al., 2018)

Human BMMSCs	Nano-TiO2 surfaces	1.3 ms-long, 75 Hz	2	Trapezoidal	10 min/day	BIOSTIM, Igea, Carpi, Italy	(Bloise et al., 2018)

**Table 3:** The table summarizes the in vitro and in vivo studies on the effects of PEMF stimulation on osteoblastic primary cells and cell lines on polymer-based biomaterials. Studies are listed in chronological order.

**Experimental model**	**Biomaterial**	**PEMF**	**Field Intensity (mT)**	**PEMF waveform**	**Exposure**	**PEMF Generator**	**Reference**
Human osteosarcoma MG-63 cells	poly-methyl methacrylate (PMMA) scaffolds or PMMA-alpha Tricalcium Phosphate (*α*-TCP) composite scaffolds	1.3 ms-long, 75 Hz	2.3	Trapezoidal	12 hours/day for 3 days	Igea, Carpi, Italy	(Torricelli et al., 2003)

Primary rat calvaria osteoblasts	Porous poly(DL-lactic-co-glycolic acid) (PLGA) scaffolds	300 *μ*s-long pulses at 7.5 Hz	0.13, 0.24 or 0.32	Rectangular	2 or 8 hours/day	PIC/16C54 series, Microchip Technology Inc., AZ	(Tsai et al., 2007)

7F2+ RAW 264.7	3D chitosan scaffolds	1.3 ms-long, 75 Hz	1.5	Trapezoidal	9 hours	BIOSTIM, Igea, Carpi, Italy	(Lin and Lin, 2011)

Human osteosarcoma Saos-2 cells	Methacrylamide-modified gelatin type B scaffolds	1.3 ms pulses at 75 Hz	2	Trapezoidal	24 hours/day for 22 days	BIOSTIM, Igea, Carpi, Italy	(Fassina et al., 2012)

Osteochondral defects in rabbit medial femoral condyles.	Commercially available equine collagen scaffolds with or w/o BMC	1.3 ms-long, 75 Hz	1.5	Trapezoidal	4 hours/day for 40 days	I-ONE, Igea, Carpi, Italy	(Veronesi et al., 2015)

Rat calvaria defects	Commercially available collagen sponges loaded with 2.5-10 *μ*g rhBMP-2	12 *μ*s pulses, 60 Hz	1	n/a	8 hours/day for 5 days	In-house built	(Yang et al., 2015)

Human adipose tissue-derived stem cells	Electrospun poly(caprolactone) nanofibrous scaffolds	50 Hz	1	n/a	6 hours/day for up to 21 days	n/a	(Arjmand et al., 2018)

## Data Availability

The data that were mentioned in this review are from previously reported studies and datasets, which have been cited. Please see the reference list and Tables 1–3.

## References

[1] Li J. J., Ebied M., Xu J., Zreiqat H. (2018). Current approaches to bone tissue engineering: the interface between biology and engineering. *Advanced Healthcare Materials*.

[2] Wang W., Yeung K. W. K. (2017). Bone grafts and biomaterials substitutes for bone defect repair: a review. *Bioactive Materials*.

[3] Shanbhag S., Pandis N., Mustafa K., Nyengaard J. R., Stavropoulos A. (2018). Bone tissue engineering in oral peri-implant defects in preclinical in vivo research: A systematic review and meta-analysis. *Journal of Tissue Engineering and Regenerative Medicine*.

[4] Rupp F., Liang L., Geis-Gerstorfer J., Scheideler L., Hüttig F. (2018). Surface characteristics of dental implants: A review. *Dental Materials*.

[5] Pearlin, Nayak S., Manivasagam G., Sen D. (2018). Progress of regenerative therapy in orthopedics. *Current Osteoporosis Reports*.

[6] Li Z., Müller R., Ruffoni D. (2017). Bone remodeling and mechanobiology around implants: Insights from small animal imaging. *Journal of Orthopaedic Research*.

[7] Kuttappan S., Mathew D., Nair M. B. (2016). Biomimetic composite scaffolds containing bioceramics and collagen/gelatin for bone tissue engineering - A mini review. *International Journal of Biological Macromolecules*.

[8] Dorozhkin S. V. (2013). Calcium orthophosphate-based bioceramics. *Materials *.

[9] Eliaz N., Metoki N. (2017). Calcium Phosphate Bioceramics: A Review of Their History, Structure, Properties, Coating Technologies and Biomedical Applications. *Materials *.

[10] Murphy W. L., McDevitt T. C., Engler A. J. (2014). Materials as stem cell regulators. *Nature Materials*.

[11] Kumar A., Placone J. K., Engler A. J. (2017). Understanding the extracellular forces that determine cell fate and maintenance. *Development*.

[12] Nabiyouni M., Brückner T., Zhou H., Gbureck U., Bhaduri S. B. (2018). Magnesium-based bioceramics in orthopedic applications. *Acta Biomaterialia*.

[13] Begam H., Nandi S. K., Kundu B., Chanda A. (2017). Strategies for delivering bone morphogenetic protein for bone healing. *Materials Science and Engineering C: Materials for Biological Applications*.

[14] Ribeiro C., Sencadas V., Correia D. M., Lanceros-Méndez S. (2015). Piezoelectric polymers as biomaterials for tissue engineering applications. *Colloids and Surfaces B: Biointerfaces*.

[15] Jahan K., Tabrizian M. (2016). Composite biopolymers for bone regeneration enhancement in bony defects. *Biomaterials Science*.

[16] Gibbs D. M. R., Black C. R. M., Dawson J. I., Oreffo R. O. C. (2016). A review of hydrogel use in fracture healing and bone regeneration. *Journal of Tissue Engineering and Regenerative Medicine*.

[17] Arakawa C., Ng R., Tan S., Kim S., Wu B., Lee M. (2017). Photopolymerizable chitosan–collagen hydrogels for bone tissue engineering. *Journal of Tissue Engineering and Regenerative Medicine*.

[18] Turnbull G., Clarke J., Picard F. (2018). 3D bioactive composite scaffolds for bone tissue engineering. *Bioactive Materials*.

[19] Martins R., Cestari T. M., Arantes R. V. N. (2018). Osseointegration of zirconia and titanium implants in a rabbit tibiae model evaluated by microtomography, histomorphometry and fluorochrome labeling analyses. *Journal of Periodontal Research*.

[20] Sivaraman K., Chopra A., Narayan A. I., Balakrishnan D. (2018). Is zirconia a viable alternative to titanium for oral implant? A critical review. *Journal of Prosthodontic Research*.

[21] Bosshardt D. D., Chappuis V., Buser D. (2017). Osseointegration of titanium, titanium alloy and zirconia dental implants: current knowledge and open questions. *Periodontology 2000*.

[22] Kellesarian S. V., Malignaggi V. R., Kellesarian T. V., Bashir Ahmed H., Javed F. (2018). Does incorporating collagen and chondroitin sulfate matrix in implant surfaces enhance osseointegration? A systematic review and meta-analysis. *International Journal of Oral and Maxillofacial Surgery*.

[23] Rao S. H., Harini B., Shadamarshan R. P. K., Balagangadharan K., Selvamurugan N. (2018). Natural and synthetic polymers/bioceramics/bioactive compounds-mediated cell signalling in bone tissue engineering. *International Journal of Biological Macromolecules*.

[24] Iskander M. F. (2013). *Electromagnetic Fields and Waves*.

[25] Birks L. E., Struchen B., Eeftens M. (2018). Spatial and temporal variability of personal environmental exposure to radio frequency electromagnetic fields in children in Europe. *Environment International*.

[26] Wertheimer N., Leeper E. (1979). Electrical wiring configurations and childhood cancer. *American Journal of Epidemiology*.

[27] Wertheimer N., Leeper E. (1982). Adult cancer related to electrical wires near the home. *International Journal of Epidemiology*.

[28] Robinette C. D., Silverman C., Jablon S. (1980). Effects upon health of occupational exposure to microwave radiation (radar). *American Journal of Epidemiology*.

[29] Valberg P. A., Kavet R., Rafferty C. N. (1997). Can low-level 50/60 hz electric and magnetic fields cause biological effects?. *Journal of Radiation Research*.

[30] Pall M. L. (2016). Microwave frequency electromagnetic fields (EMFs) produce widespread neuropsychiatric effects including depression. *Journal of Chemical Neuroanatomy*.

[31] Pall M. L. (2018). Wi-Fi is an important threat to human health. *Environmental Research*.

[32] Hug K., Röösli M. (2012). Therapeutic effects of whole-body devices applying pulsed electromagnetic fields (PEMF): A systematic literature review. *Bioelectromagnetics*.

[33] Zhang X., Zhang J., Qu X., Wen J. (2007). Effects of Different Extremely Low-Frequency Electromagnetic Fields on Osteoblasts. *Electromagnetic Biology and Medicine*.

[34] Pchelintseva E., Djamgoz M. B. A. (2017). Mesenchymal stem cell differentiation: Control by calcium-activated potassium channels. *Journal of Cellular Physiology*.

[35] Zhang X., Liu X., Pan L., Lee I. (2010). Magnetic fields at extremely low-frequency (50 Hz, 0.8 mT) can induce the uptake of intracellular calcium levels in osteoblasts. *Biochemical and Biophysical Research Communications*.

[36] Tong J., Sun L., Zhu B. (2017). Pulsed electromagnetic fields promote the proliferation and differentiation of osteoblasts by reinforcing intracellular calcium transients. *Bioelectromagnetics*.

[37] Kuan-Jung Li J., Cheng-An Lin J., Liu H. (2006). Comparison of ultrasound and electromagnetic field effects on osteoblast growth. *Ultrasound in Medicine & Biology*.

[38] Wu S., Yu Q., Lai A., Tian J. (2018). Pulsed electromagnetic field induces Ca2+ -dependent osteoblastogenesis in C3H10T1/2 mesenchymal cells through the Wnt-Ca 2+ /Wnt-*β*-catenin signaling pathway. *Biochemical and Biophysical Research Communications*.

[39] Varani K., Vincenzi F., Ravani A. (2017). Adenosine receptors as a biological pathway for the anti-inflammatory and beneficial effects of low frequency low energy pulsed electromagnetic fields. *Mediators of Inflammation*.

[40] Yan J., Zhou J., Ma H. (2015). Pulsed electromagnetic fields promote osteoblast mineralization and maturation needing the existence of primary cilia. *Molecular and Cellular Endocrinology*.

[41] Xie Y.-F., Shi W.-G., Zhou J. (2016). Pulsed electromagnetic fields stimulate osteogenic differentiation and maturation of osteoblasts by upregulating the expression of BMPRII localized at the base of primary cilium. *Bone*.

[42] Ehnert S., Fentz A., Schreiner A. (2017). Extremely low frequency pulsed electromagnetic fields cause antioxidative defense mechanisms in human osteoblasts via induction of •O2− and H2O2. *Scientific Reports*.

[43] Lohmann C. H., Schwartz Z., Liu Y. (2000). Pulsed electromagnetic field stimulation of MG63 osteoblast-like cells affects differentiation and local factor production. *Journal of Orthopaedic Research*.

[44] Sakai Y., Patterson T. E., Ibiwoye M. O. (2006). Exposure of mouse preosteoblasts to pulsed electromagnetic fields reduces the amount of mature, type I collagen in the extracellular matrix. *Journal of Orthopaedic Research*.

[45] Bodamyali T., Bhatt B., Hughes F. J. (1998). Pulsed electromagnetic fields simultaneously induce osteogenesis and upregulate transcription of bone morphogenetic proteins 2 and 4 in rat osteoblasts in vitro. *Biochemical and Biophysical Research Communications*.

[46] Wang Y., Pu X., Shi W. (2018). Pulsed electromagnetic fields promote bone formation by activating the sAC-cAMP-PKA-CREB signaling pathway. *Journal of Cellular Physiology*.

[47] Daish C., Blanchard R., Fox K., Pivonka P., Pirogova E. (2018). The Application of Pulsed Electromagnetic Fields (PEMFs) for Bone Fracture Repair: Past and Perspective Findings. *Annals of Biomedical Engineering*.

[48] Huegel J., Choi D. S., Nuss C. A. (2018). Effects of pulsed electromagnetic field therapy at different frequencies and durations on rotator cuff tendon-to-bone healing in a rat model. *Journal of Shoulder and Elbow Surgery*.

[49] Bilgin H. M., Çelik F., Gem M. (2017). Effects of local vibration and pulsed electromagnetic field on bone fracture: A comparative study. *Bioelectromagnetics*.

[50] Sarker A. B., Nashimuddin A. N., Islam K. M. (1993). Effect of PEMF on fresh fracture-healing in rat tibia. *Bangladesh Medical Research Council Bulletin*.

[51] Taylor K. F., Inoue N., Rafiee B., Tis J. E., McHale K. A., Chao E. Y. S. (2006). Effect of pulsed electromagnetic fields on maturation of regenerate bone in a rabbit limb lengthening model. *Journal of Orthopaedic Research*.

[52] Fredericks D. C., Nepola J. V., Baker J. T., Abbott J., Simon B. (2000). Effects of pulsed electromagnetic fields on bone healing in a rabbit tibial osteotomy model. *Journal of Orthopaedic Trauma*.

[53] Midura R. J., Ibiwoye M. O., Powell K. A. (2005). Pulsed electromagnetic field treatments enhance the healing of fibular osteotomies. *Journal of Orthopaedic Research*.

[54] Landry P. S., Sadasivan K. K., Marino A. A., Albright J. A. (1997). Electromagnetic Fields Can Affect Osteogenesis by Increasing the Rate of Differentiation. *Clinical Orthopaedics and Related Research*.

[55] Takano-Yamamoto T., Kawakami M., Sakuda M. (1992). Effect of a pulsing electromagnetic field on demineralized bone-matrix-induced bone formation in a bony defect in the premaxilla of rats. *Journal of Dental Research*.

[56] Kapi E., Bozkurt M., Selcuk C. T. (2015). Comparison of effects of pulsed electromagnetic field stimulation on platelet-rich plasma and bone marrow stromal stem cell using rat zygomatic bone defect model. *Annals of Plastic Surgery*.

[57] Liu C., Zhang Y., Fu T. (2017). Effects of electromagnetic fields on bone loss in hyperthyroidism rat model. *Bioelectromagnetics*.

[58] Jing D., Shen G., Huang J. (2010). Circadian rhythm affects the preventive role of pulsed electromagnetic fields on ovariectomy-induced osteoporosis in rats. *Bone*.

[59] Jiang Y., Gou H., Wang S., Zhu J., Tian S., Yu L. (2016). Effect of pulsed electromagnetic field on bone formation and lipid metabolism of glucocorticoid-induced osteoporosis rats through canonical wnt signaling pathway. *Evidence-Based Complementary and Alternative Medicine*.

[60] Zhou J., He H., Yang L. (2012). Effects of pulsed electromagnetic fields on bone mass and Wnt/*β*-catenin signaling pathway in ovariectomized rats. *Archives of Medical Research*.

[61] Zhou J., Chen S., Guo H. (2013). Pulsed electromagnetic field stimulates osteoprotegerin and reduces RANKL expression in ovariectomized rats. *Rheumatology International*.

[62] Zhou J., Liao Y., Zeng Y., Xie H., Fu C., Li N. (2017). Effect of intervention initiation timing of pulsed electromagnetic field on ovariectomy-induced osteoporosis in rats. *Bioelectromagnetics*.

[63] Zhou J., Liao Y., Xie H. (2017). Effects of combined treatment with ibandronate and pulsed electromagnetic field on ovariectomy-induced osteoporosis in rats. *Bioelectromagnetics*.

[64] Jing D., Li F., Jiang M. (2013). Pulsed electromagnetic fields improve bone microstructure and strength in ovariectomized rats through a Wnt/Lrp5/*β*-catenin signaling-associated mechanism. *PLoS ONE*.

[65] Lei T., Liang Z., Li F. (2018). Pulsed electromagnetic fields (PEMF) attenuate changes in vertebral bone mass, architecture and strength in ovariectomized mice. *Bone*.

[66] Chang K., Chang W. H.-S. (2003). Pulsed electromagnetic fields prevent osteoporosis in an ovariectomized female rat model: a prostaglandin E2-associated process. *Bioelectromagnetics*.

[67] Jing D., Cai J., Wu Y. (2014). Pulsed electromagnetic fields partially preserve bone mass, microarchitecture, and strength by promoting bone formation in hindlimb-suspended rats. *Journal of Bone and Mineral Research*.

[68] Li B., Bi J., Li W. (2017). Effects of pulsed electromagnetic fields on histomorphometry and osteocalcin in disuse osteoporosis rats. *Technology and Health Care*.

[69] Shen W.-W., Zhao J.-H. (2010). Pulsed electromagnetic fields stimulation affects BMD and local factor production of rats with disuse osteoporosis. *Bioelectromagnetics*.

[70] Li J., Zeng Z., Zhao Y. (2017). Effects of low-intensity pulsed electromagnetic fields on bone microarchitecture, mechanical strength and bone turnover in type 2 diabetic db/db mice. *Scientific Reports*.

[71] Androjna C., Fort B., Zborowski M., Midura R. J. (2014). Pulsed electromagnetic field treatment enhances healing callus biomechanical properties in an animal model of osteoporotic fracture. *Bioelectromagnetics*.

[72] Yang X., He H., Zhou Y. (2017). Pulsed electromagnetic field at different stages of knee osteoarthritis in rats induced by low-dose monosodium iodoacetate: Effect on subchondral trabecular bone microarchitecture and cartilage degradation. *Bioelectromagnetics*.

[73] Canè V., Botti P., Farneti D., Soana S. (1991). Electromagnetic stimulation of bone repair: A histomorphometric study. *Journal of Orthopaedic Research*.

[74] Cane V., Botti P., Soana S. (1993). Pulsed magnetic fields improve osteoblast activity during the repair of an experimental osseous defect. *Journal of Orthopaedic Research*.

[75] Garland D. E., Adkins R. H., Matsuno N. N., Stewart C. A. (1999). The effect of pulsed electromagnetic fields on osteoporosis at the knee in individuals with spinal cord injury. *The Journal of Spinal Cord Medicine*.

[76] Tabrah F. L., Ross P., Hoffmeier M., Gilbert F. (1998). Clinical Report on Long-Term Bone Density after Short-Term EMF Application. *Bioelectromagnetics*.

[77] Tabrah F., Hoffmeier M., Gilbert F., Batkin S., Bassett C. A. L. (1990). Bone density changes in osteoporosis‐prone women exposed to pulsed electromagnetic fields (PEMFs). *Journal of Bone and Mineral Research*.

[78] Bassett C. A., Pilla A. A., Pawluk R. J. (1977). A non-operative salvage of surgically-resistant pseudarthroses and non-unions by pulsing electromagnetic fields. *Clinical Orthopaedics and Related Research*.

[79] Bassett C. A., Mitchell S. N., Gaston S. R. (1981). Treatment of ununited tibial diaphyseal fractures with pulsing electromagnetic fields.. *The Journal of Bone & Joint Surgery*.

[80] Simonis R. B., Parnell E. J., Ray P. S., Peacock J. L. (2003). Electrical treatment of tibial non-union: A prospective, randomised, double-blind trial. *Injury*.

[81] Lazovic M., Kocic M., Dimitrijevic L., Stankovic I., Spalevic M., Ciric T. (2012). Pulsed electromagnetic field during cast immobilization in postmenopausal women with Colles’ fracture. *Srpski Arhiv za Celokupno Lekarstvo*.

[82] Cheing G. L. Y., Wan J. W. H., Kai Lo S. (2005). Ice and pulsed electromagnetic field to reduce pain and swelling after distal radius fractures. *Journal of Rehabilitation Medicine*.

[83] Hannemann P. F., Essers B. A., Schots J. P., Dullaert K., Poeze M., Brink P. R. (2015). Functional outcome and cost-effectiveness of pulsed electromagnetic fields in the treatment of acute scaphoid fractures: a cost-utility analysis. *BMC Musculoskeletal Disorders*.

[84] Faldini C., Cadossi M., Luciani D., Betti E., Chiarello E., Giannini S. (2010). Electromagnetic bone growth stimulation in patients with femoral neck fractures treated with screws: Prospective randomized double-blind study. *Current Orthopaedic Practice*.

[85] Adie S., Harris I. A., Naylor J. M. (2011). Pulsed Electromagnetic Field Stimulation for Acute Tibial Shaft Fractures. *The Journal of Bone and Joint Surgery-American Volume*.

[86] Assiotis A., Sachinis N. P., Chalidis B. E. (2012). Pulsed electromagnetic fields for the treatment of tibial delayed unions and nonunions. A prospective clinical study and review of the literature. *Journal of Orthopaedic Surgery and Research*.

[87] Shi H., Xiong J., Chen Y. (2013). Early application of pulsed electromagnetic field in the treatment of postoperative delayed union of long-bone fractures: a prospective randomized controlled study. *BMC Musculoskeletal Disorders*.

[88] Streit A., Watson B. C., Granata J. D. (2016). Effect on Clinical Outcome and Growth Factor Synthesis With Adjunctive Use of Pulsed Electromagnetic Fields for Fifth Metatarsal Nonunion Fracture. *Foot & Ankle International*.

[89] Refai H., Radwan D., Hassanien N. (2014). Radiodensitometric Assessment of the Effect of Pulsed Electromagnetic Field Stimulation Versus Low Intensity Laser Irradiation on Mandibular Fracture Repair: A Preliminary Clinical Trial. *Journal of Maxillofacial and Oral Surgery*.

[90] Abdelrahim A., Hassanein H. R., Dahaba M. (2011). Effect of pulsed electromagnetic field on healing of mandibular fracture: a preliminary clinical study. *Journal of Oral and Maxillofacial Surgery*.

[91] Barker A. T., Dixon R. A., Sharrard W. J. W., Sutcliffe M. L. (1984). Pulsed Magnetic Field Therapy for Tibial Non-Union. Interim Results of a Double-Blind Trial. *The Lancet*.

[92] Scott G., King J. B. (1994). A prospective, double-blind trial of electrical capacitive coupling in the treatment of non-union of long bones. *The Journal of Bone & Joint Surgery*.

[93] Sharrard W. (1990). A double-blind trial of pulsed electromagnetic fields for delayed union of tibial fractures. *The Journal of Bone & Joint Surgery*.

[94] Liu C., Yu J., Yang Y. (2013). Effect of 1 mT sinusoidal electromagnetic fields on proliferation and osteogenic differentiation of rat bone marrow mesenchymal stromal cells. *Bioelectromagnetics*.

[95] Li K., Ma S., Li Y. (2014). Effects of PEMF exposure at different pulses on osteogenesis of MC3T3-E1 cells. *Archives of Oral Biology*.

[96] Markov M. S. (2007). Magnetic Field Therapy: A Review. *Electromagnetic Biology and Medicine*.

[97] Lei T., Li F., Liang Z. (2017). Effects of four kinds of electromagnetic fields (EMF) with different frequency spectrum bands on ovariectomized osteoporosis in mice. *Scientific Reports*.

[98] Zhou J., Wang J.-Q., Ge B.-F. (2014). Different electromagnetic field waveforms have different effects on proliferation, differentiation and mineralization of osteoblasts in vitro. *Bioelectromagnetics*.

[99] Hubbard D. K., Dennis R. (2012). Pain relief and tissue healing using pemf therapy: a review of stimulation waveform effects. *Asia Health Care Journal*.

[100] Griffin X. L., Costa M. L., Parsons N., Smith N. (2011). Electromagnetic field stimulation for treating delayed union or non-union of long bone fractures in adults. *Cochrane Database of Systematic Reviews*.

[101] Handoll H. H., Elliott J. (2015). Rehabilitation for distal radial fractures in adults. *Cochrane Database of Systematic Reviews*.

[102] Hannemann P. F. W., Mommers E. H. H., Schots J. P. M., Brink P. R. G., Poeze M. (2014). The effects of low-intensity pulsed ultrasound and pulsed electromagnetic fields bone growth stimulation in acute fractures: a systematic review and meta-analysis of randomized controlled trials. *Archives of Orthopaedic and Trauma Surgery*.

[103] Massari L., Caruso G., Sollazzo V. (2009). Pulsed electromagnetic fields and low intensity pulsed ultrasound in bone tissue. *Clinical Cases in Mineral Bone Metabolism*.

[104] Martino C. F., Belchenko D., Ferguson V., Nielsen-Preiss S., Qi H. J. (2008). The effects of pulsed electromagnetic fields on the cellular activity of SaOS-2 cells. *Bioelectromagnetics*.

[105] Hannay G., Leavesley D., Pearcy M. (2005). Timing of pulsed electromagnetic field stimulation does not affect the promotion of bone cell development. *Bioelectromagnetics*.

[106] Noriega-Luna B., Sabanero M., Sosa M., Avila-Rodriguez M. (2011). Influence of pulsed magnetic fields on the morphology of bone cells in early stages of growth. *Micron*.

[107] Sollazzo V., Palmieri A., Pezzetti F., Massari L., Carinci F. (2010). Effects of pulsed electromagnetic fields on human osteoblastlike cells (MG-63): a pilot study. *Clinical Orthopaedics and Related Research*.

[108] De Mattei M., Gagliano N., Moscheni C. (2005). Changes in polyamines, c-myc and c-fos gene expression in osteoblast-like cells exposed to pulsed electromagnetic fields. *Bioelectromagnetics*.

[109] Soda A., Ikehara T., Kinouchi Y., Yoshizaki K. (2008). Effect of exposure to an extremely low frequency-electromagnetic field on the cellular collagen with respect to signaling pathways in osteoblast-like cells. *The Journal of Medical Investigation*.

[110] Zhai M., Jing D., Tong S. (2016). Pulsed electromagnetic fields promote in vitro osteoblastogenesis through a Wnt/*β*-catenin signaling-associated mechanism. *Bioelectromagnetics*.

[111] Patterson T. E., Sakai Y., Grabiner M. D. (2006). Exposure of murine cells to pulsed electromagnetic fields rapidly activates the mTOR signaling pathway. *Bioelectromagnetics*.

[112] Diniz P., Soejima K., Ito G. (2002). Nitric oxide mediates the effects of pulsed electromagnetic field stimulation on the osteoblast proliferation and differentiation. *Nitric Oxide: Biology and Chemistry*.

[113] Diniz P., Shomura K., Soejima K., Ito G. (2002). Effects of Pulsed Electromagnetic Field (PEMF) Stimulation on Bone Tissue Like Formation Are Dependent on the Maturation Stages of the Osteoblasts. *Bioelectromagnetics*.

[114] Lin C.-C., Lin R.-W., Chang C.-W., Wang G.-J., Lai K.-A. (2015). Single-pulsed electromagnetic field therapy increases osteogenic differentiation through Wnt signaling pathway and sclerostin downregulation. *Bioelectromagnetics*.

[115] Selvamurugan N., Kwok S., Vasilov A., Jefcoat S. C., Partridge N. C. (2007). Effects of BMP-2 and pulsed electromagnetic field (PEMF) on rat primary osteoblastic cell proliferation and gene expression. *Journal of Orthopaedic Research*.

[116] Hopper R. A., Verhalen J. P., Tepper O. T. (2009). Osteoblasts stimulated with pulsed electromagnetic fields increase HUVEC proliferation via a VEGF-A independent mechanism. *Bioelectromagnetics*.

[117] Barnaba S., Papalia R., Ruzzini L., Sgambato A., Maffulli N., Denaro V. (2013). Effect of pulsed electromagnetic fields on human osteoblast cultures. *Physiotherapy Research International*.

[118] Ehnert S., van Griensven M., Unger M. (2018). Co-Culture with Human Osteoblasts and Exposure to Extremely Low Frequency Pulsed Electromagnetic Fields Improve Osteogenic Differentiation of Human Adipose-Derived Mesenchymal Stem Cells. *International Journal of Molecular Sciences*.

[119] Ehnert S., Falldorf K., Fentz A.-K. (2015). Primary human osteoblasts with reduced alkaline phosphatase and matrix mineralization baseline capacity are responsive to extremely low frequency pulsed electromagnetic field exposure - Clinical implication possible. *Bone Reports*.

[120] Ceccarelli G., Bloise N., Mantelli M. (2013). A comparative analysis of the in vitro effects of pulsed electromagnetic field treatment on osteogenic differentiation of two different mesenchymal cell lineages. *BioResearch Open Access*.

[121] Ferroni L., Tocco I., De Pieri A. (2016). Pulsed magnetic therapy increases osteogenic differentiation of mesenchymal stem cells only if they are pre-committed. *Life Sciences*.

[122] Yin Y., Chen P., Yu Q., Peng Y., Zhu Z., Tian J. (2018). The Effects of a Pulsed Electromagnetic Field on the Proliferation and Osteogenic Differentiation of Human Adipose-Derived Stem Cells. *Medical Science Monitor*.

[123] Fu Y.-C., Lin C.-C., Chang J.-K. (2014). A novel single pulsed electromagnetic field stimulates osteogenesis of bone marrow mesenchymal stem cells and bone repair. *PLoS ONE*.

[124] Petecchia L., Sbrana F., Utzeri R. (2015). Electro-magnetic field promotes osteogenic differentiation of BM-hMSCs through a selective action on Ca^2+^-related mechanisms. *Scientific Reports*.

[125] Jazayeri M., Shokrgozar M. A., Haghighipour N., Bolouri B., Mirahmadi F., Farokhi M. (2017). Effects of electromagnetic stimulation on gene expression of mesenchymal stem cells and repair of bone lesions. *Cell*.

[126] Tsai M.-T., Li W.-J., Tuan R. S., Chang W. H. (2009). Modulation of osteogenesis in human mesenchymal stem cells by specific pulsed electromagnetic field stimulation. *Journal of Orthopaedic Research*.

[127] Sun L.-Y., Hsieh D.-K., Lin P.-C., Chiu H.-T., Chiou T.-W. (2009). Pulsed electromagnetic fields accelerate proliferation and osteogenic gene expression in human bone marrow mesenchymal stem cells during osteogenic differentiation. *Bioelectromagnetics*.

[128] Sun L.-Y., Hsieh D.-K., Yu T.-C. (2009). Effect of pulsed electromagnetic field on the proliferation and differentiation potential of human bone marrow mesenchymal stem cells. *Bioelectromagnetics*.

[129] Jansen J. H. W., van der Jagt O. P., Punt B. J. (2010). Stimulation of osteogenic differentiation in human osteoprogenitor cells by pulsed electromagnetic fields: an in vitro study. *BMC Musculoskeletal Disorders*.

[130] Kaivosoja E., Sariola V., Chen Y., Konttinen Y. T. (2015). The effect of pulsed electromagnetic fields and dehydroepiandrosterone on viability and osteo-induction of human mesenchymal stem cells. *Journal of Tissue Engineering and Regenerative Medicine*.

[131] Selvamurugan N., He Z., Rifkin D., Dabovic B., Partridge N. C. (2017). Pulsed Electromagnetic Field Regulates MicroRNA 21 Expression to Activate TGF- *β* Signaling in Human Bone Marrow Stromal Cells to Enhance Osteoblast Differentiation. *Stem Cells International*.

[132] He Z., Selvamurugan N., Warshaw J., Partridge N. C. (2018). Pulsed electromagnetic fields inhibit human osteoclast formation and gene expression via osteoblasts. *Bone*.

[133] Esposito M., Lucariello A., Riccio I., Riccio V., Esposito V., Riccardi G. (2012). Differentiation of human osteoprogenitor cells increases after treatment with pulsed electromagnetic fields. *In Vivo (Brooklyn)*.

[134] De Mattei M., Caruso A., Traina G. C., Pezzetti F., Baroni T., Sollazzo V. (1999). Correlation between pulsed electromagnetic fields exposure time and cell proliferation increase in human osteosarcoma cell lines and human normal osteoblast cells in vitro. *Bioelectromagnetics*.

[135] Lin H.-Y., Lin Y.-J. (2011). In vitro effects of low frequency electromagnetic fields on osteoblast proliferation and maturation in an inflammatory environment. *Bioelectromagnetics*.

[136] Wang J., Tang N., Xiao Q. (2015). Pulsed electromagnetic field may accelerate in vitro endochondral ossification. *Bioelectromagnetics*.

[137] Bagheri L., Pellati A., Rizzo P. (2017). Notch pathway is active during osteogenic differentiation of human bone marrow mesenchymal stem cells induced by pulsed electromagnetic fields. *Journal of Tissue Engineering and Regenerative Medicine*.

[138] Luben R. A., Cain C. D., Chen M. C.-Y., Rosen D. M., Adey W. R. (1982). Effects of electromagnetic stimuli on bone and bone cells in vitro: Inhibition of responses to parathyroid hormone by low-energy low-frequency fields. *Proceedings of the National Acadamy of Sciences of the United States of America*.

[139] Lohmann C. H., Schwartz Z., Liu Y. (2003). Pulsed electromagnetic fields affect phenotype and connexin 43 protein expression in MLO-Y4 osteocyte-like cells and ROS 17/2.8 osteoblast-like cells. *Journal of Orthopaedic Research*.

[140] Shimizu T., Zerwekh J. E., Videman T. (1988). Bone ingrowth into porous calcium phosphate ceramics: Influence of pulsing electromagnetic field. *Journal of Orthopaedic Research*.

[141] Ottani V., Raspanti M., Martini D. (2002). Electromagnetic stimulation on the bone growth using backscattered electron imaging. *Micron*.

[142] Fini M., Cadossi R. (2002). The effect of pulsed electromagnetic fields on the osteointegration of hydroxyapatite implants in cancellous bone: a morphologic and microstructural in vivo study. *Journal of Orthopaedic Research*.

[143] Fini M., Giavaresi G., Giardino R., Cavani F., Cadossi R. (2006). Histomorphometric and mechanical analysis of the hydroxyapatite-bone interface after electromagnetic stimulation. *The Journal of Bone & Joint Surgery (British Volume)*.

[144] Fassina L., Saino E., Sbarra M. S. (2010). In vitro electromagnetically stimulated SAOS-2 osteoblasts inside porous hydroxyapatite. *Journal of Biomedical Materials Research Part A*.

[145] Schwartz Z., Simon B. J., Duran M. A., Barabino G., Chaudhri R., Boyan B. D. (2008). Pulsed electromagnetic fields enhance BMP-2 dependent osteoblastic differentiation of human mesenchymal stem cells. *Journal of Orthopaedic Research*.

[146] Schwartz Z., Fisher M., Lohmann C. H., Simon B. J., Boyan B. D. (2009). Osteoprotegerin (OPG) production by cells in the osteoblast lineage is regulated by pulsed electromagnetic fields in cultures grown on calcium phosphate substrates. *Annals of Biomedical Engineering*.

[147] Martin T. J., Sims N. A. (2015). RANKL/OPG; Critical role in bone physiology. *Reviews in Endocrine and Metabolic Disorders*.

[148] Simonet W. S., Lacey D. L., Dunstan C. R. (1997). Osteoprotegerin: a novel secreted protein involved in the regulation of bone density. *Cell*.

[149] Yasuda H., Shima N., Nakagawa N. Osteoclast differentiation factor is a ligand for osteoprotegerin/osteoclastogenesis-inhibitory factor and is identical to TRANCE/RANKL.

[150] Tsuda E., Goto M., Mochizuki S.-I. (1997). Isolation of a novel cytokine from human fibroblasts that specifically inhibits osteoclastogenesis. *Biochemical and Biophysical Research Communications*.

[151] Chang K., Chang W. H.-S., Huang S., Huang S., Shih C. (2005). Pulsed electromagnetic fields stimulation affects osteoclast formation by modulation of osteoprotegerin, RANK ligand and macrophage colony-stimulating factor. *Journal of Orthopaedic Research*.

[152] Chang W. H.-S., Chen L.-T., Sun J.-S., Lin F.-H. (2004). Effect of pulse-burst electromagnetic field stimulation on osteoblast cell activities. *Bioelectromagnetics*.

[153] Spadaro J. A., Albanese S. A., Chase S. E. (1990). Electromagnetic effects on bone formation at implants in the medullary canal in rabbits. *Journal of Orthopaedic Research*.

[154] Fassina L., Saino E., Visai L. (2008). Electromagnetic enhancement of a culture of human SAOS-2 osteoblasts seeded onto titanium fiber-mesh scaffolds. *Journal of Biomedical Materials Research Part A*.

[155] Fassina L., Saino E., Visai L., Magenes G. Electromagnetically enhanced coating of a sintered titanium grid with human SAOS-2 osteoblasts and extracellular matrix.

[156] Wang J., An Y., Li F. (2014). The effects of pulsed electromagnetic field on the functions of osteoblasts on implant surfaces with different topographies. *Acta Biomaterialia*.

[157] Bloise N., Petecchia L., Ceccarelli G. (2018). The effect of pulsed electromagnetic field exposure on osteoinduction of human mesenchymal stem cells cultured on nano-TiO2 surfaces. *PLoS ONE*.

[158] Carbone R., Marangi I., Zanardi A. (2006). Biocompatibility of cluster-assembled nanostructured TiO_2_ with primary and cancer cells. *Biomaterials*.

[159] Vercellino M., Ceccarelli G., Cristofaro F. (2016). Nanostructured TiO_2_ surfaces promote human bone marrow mesenchymal stem cells differentiation to osteoblasts. *Nanomaterials*.

[160] Atalay B., Aybar B., Ergüven M. (2013). The Effects of Pulsed Electromagnetic Field (PEMF) on Osteoblast-Like Cells Cultured on Titanium and Titanium-Zirconium Surfaces. *The Journal of Craniofacial Surgery*.

[161] Jing D., Zhai M., Tong S. (2016). Pulsed electromagnetic fields promote osteogenesis and osseointegration of porous titanium implants in bone defect repair through a Wnt/*β*-catenin signaling-associated mechanism. *Scientific Reports*.

[162] Buzzá E. P., Shibli J. A., Barbeiro R. H., Barbosa J. R. D. A. (2003). Effects of electromagnetic field on bone healing around commercially pure titanium surface: Histologic and mechanical study in rabbits. *Implant Dentistry*.

[163] Do Nascimento C., Issa J. P. M., Da Silva Mello A. S., De Albuquerque Junior R. F. (2012). Effect of electromagnetic field on bone regeneration around dental implants after immediate placement in the dog mandible: A pilot study. *Gerodontology*.

[164] Matsumoto H., Ochi M., Abiko Y., Hirose Y., Kaku T., Sakaguchi K. (2000). Pulsed electromagnetic fields promote bone formation around dental implants inserted into the femur of rabbits. *Clinical Oral Implants Research*.

[165] Özen J., Atay A., Orucß S., Dalkiz M., Beydemir B., Develi S. (2004). Evaluation of pulsed electromagnetic fields on bone healing after implant placement in the rabbit mandibular model. *Turkish Journal of Medical Sciences*.

[166] Akca K., Sarac E., Baysal U., Fanuscu M., Chang T., Cehreli M. (2007). Micro-morphologic changes around biophysically-stimulated titanium implants in ovariectomized rats. *Head & Face Medicine*.

[167] Grana D. R., Marcos H. J. A., Kokubu G. A. (2018). Pulsed electromagnetic fields as adjuvant therapy in bone healing and peri-implant bone formation: an experimental study in rats. *Acta Odontologica Latinoamericana*.

[168] Barak S., Neuman M., Iezzi G., Piattelli A., Perrotti V., Gabet Y. (2016). A new device for improving dental implants anchorage: A histological and micro-computed tomography study in the rabbit. *Clinical Oral Implants Research*.

[169] Chan A. Y. Development of an intra-oral bone growth stimulator for titanium dental implants.

[170] Bambini F., Santarelli A., Putignano A. (2017). Use of supercharged cover screw as static magnetic field generator for bone healing, 2nd part: in vivo enhancement of bone regeneration in rabbits. *Journal of Biologucal Regulators and Homeostatic Agents*.

[171] Ijiri K., Matsunaga S., Fukuyama K. (1996). The effect of pulsing electromagnetic field on bone ingrowth into a porous coated implant. *Anticancer Research*.

[172] Cai J., Li W., Sun T., Li X., Luo E., Jing D. (2018). Pulsed electromagnetic fields preserve bone architecture and mechanical properties and stimulate porous implant osseointegration by promoting bone anabolism in type 1 diabetic rabbits. *Osteoporosis International*.

[173] Arjmand M., Ardeshirylajimi A., Maghsoudi H., Azadian E. (2018). Osteogenic differentiation potential of mesenchymal stem cells cultured on nanofibrous scaffold improved in the presence of pulsed electromagnetic field. *Journal of Cellular Physiology*.

[174] Tsai M.-T., Chang W. H.-S., Chang K., Hou R.-J., Wu T.-W. (2007). Pulsed electromagnetic fields affect osteoblast proliferation and differentiation in bone tissue engineering. *Bioelectromagnetics*.

[175] Schnoke M., Midura R. J. (2007). Pulsed electromagnetic fields rapidly modulate intracellular signaling events in osteoblastic cells: Comparison to parathyroid hormone and insulin. *Journal of Orthopaedic Research*.

[176] Patruno A., Amerio P., Pesce M. (2010). Extremely low frequency electromagnetic fields modulate expression of inducible nitric oxide synthase, endothelial nitric oxide synthase and cyclooxygenase-2 in the human keratinocyte cell line HaCat: Potential therapeutic effects in wound healing. *British Journal of Dermatology*.

[177] Torricelli P., Fini M., Giavaresi G., Botter R., Beruto D., Giardino R. (2003). Biomimetic PMMA-based bone substitutes: A comparativein vitro evaluation of the effects of pulsed electromagnetic field exposure. *Journal of Biomedical Materials Research Part B: Applied Biomaterials*.

[178] Veronesi F., Cadossi M., Giavaresi G. (2015). Pulsed electromagnetic fields combined with a collagenous scaffold and bone marrow concentrate enhance osteochondral regeneration: an in vivo study. *BMC Musculoskeletal Disorders*.

[179] Yang H. J., Kim R. Y., Hwang S. J. (2015). Pulsed electromagnetic fields enhance bone morphogenetic protein-2 dependent-bone regeneration. *Tissue Engineering Part: A*.

[180] Fassina L., Saino E., Visai L. (2012). Electromagnetic Stimulation to Optimize the Bone Regeneration Capacity of Gelatin-Based Cryogels. *International Journal of Immunopathology and Pharmacology*.

[181] Fassina L., Saino E., Visai L., Magenes G. Electromagnetically enhanced coating of a sintered titanium grid with human SAOS-2 osteoblasts and extracellular matrix.

[182] Silva E. D., Babo P. S., Costa-Almeida R. (2017). Multifunctional magnetic-responsive hydrogels to engineer tendon-to-bone interface. *Nanomedicine: Nanotechnology, Biology and Medicine*.

[183] Kondaveeti S., Semeano A. T., Cornejo D. R., Ulrich H., Petri D. F. (2018). Magnetic hydrogels for levodopa release and cell stimulation triggered by external magnetic field. *Colloids and Surfaces B: Biointerfaces*.

[184] Kondaveeti S., Cornejo D. R., Petri D. F. S. (2016). Alginate/magnetite hybrid beads for magnetically stimulated release of dopamine. *Colloids and Surfaces B: Biointerfaces*.

